# Training Hospitalists in Negotiations to Address Conflicts with Older Adults around Their Social Needs

**DOI:** 10.3390/geriatrics5030050

**Published:** 2020-09-14

**Authors:** Mobola Campbell, Vanessa Ramirez-Zohfeld, Anne Seltzer, Lee A. Lindquist

**Affiliations:** 1 Department of Medicine, Mayo Clinic, Jacksonville, FL 32224, USA; Campbell.Mobola@mayo.edu; 2 Feinberg School of Medicine, Northwestern University, Chicago, IL 60611, USA; vanessa-ramirez-0@northwestern.edu (V.R.-Z.); aseltzer@nm.org (A.S.)

**Keywords:** hospitalist, hospital medicine, social needs, older adults, negotiations

## Abstract

Hospitalists care for a growing population of older patients with unique social needs that can often be addressed by providing caregiver help in the home. The importance of addressing social needs is well-recognized, yet older patients sometimes refuse these services. This refusal of services may result in rehospitalization and increased morbidity for patients. We aimed to overcome this refusal of social support through an innovative workshop training hospitalists in negotiation and dispute resolution. Hospitalists at a tertiary care, urban academic medical center completed a one-hour interactive workshop on negotiation and dispute resolution focused on addressing older patients’ refusal of social services. One month post workshop, participants reported increased confidence in engaging patients and families in conflicts and felt empowered to negotiate in both their clinical practice and personal lives. Training hospitalists to negotiate with older adults needing social services is feasible and positively impacts the ability to provide geriatric care.

## 1. Introduction

Older adults constitute a rapidly growing portion of hospitalized patients and have unique social support needs that hospitalists may not be adequately trained to meet—these include support for decrease in both activities of daily living (ADL) and instrumental activities of daily living (IADL) [1,2,3]. Furthermore, older adult self-neglect and refusal of social services may negatively impact their health and ability to age-in-place in their own homes [4,5,6]. Identifying and connecting older adults with social support services may seem simple, but hospitalists frequently encounter conflicts with older adult patients and their families around refusal of care [7]. Often hospitalists lack formal training in navigating these difficult conversations and interactions [8]. We hypothesized that training hospitalists in negotiations and dispute resolution might have the potential to improve the care of older adults. Although longer term impact, such as improving quality metrics and reducing hospitalist frustration and burnout, were not tested in this study, we aimed to lay the groundwork to explore the potential for such impact in future studies.

Negotiations and dispute resolution (NDR) training is widely used in business, law, communication, and public policy sectors to work through conflicts [9]. The training generally consists of didactic sessions with exercises where participants learn and refine their negotiation skills. Within the healthcare field, while negotiation has been identified as a crucial skill, very few faculty report having the necessary skills to successfully negotiate [10]. Within graduate medical education, negotiations are typically mentioned in the setting of job contracts but not direct patient care. We hypothesized that the knowledge and skills gained from NDR training can be applied to conflicts in the clinical setting.

## 2. Materials and Methods

### 2.1. Needs Assessment

The study was conducted at an urban academic medical center in April 2019. An invitation was sent out to hospitalists and advanced practice providers (nurse practitioners and physician assistants) in the Division of Hospital Medicine and were able to attend the in-person workshop, and provided care for older adults age 65 and over. Participants also had to confirm access to the internet and an email address to participate. The study was deemed exempt by the Northwestern University Institutional Review Board.

We conducted a needs assessment of hospitalists to determine their current experience in disputes and conflicts in the clinical setting, their baseline knowledge and past experience in negotiations, and their desire to gain skills in negotiations and dispute resolution.

### 2.2. Workshop and Surveys

Based on the results of the needs assessment, we tailored the NDR workshop, which was originally modelled after Northwestern University Kellogg School of Business negotiations and dispute resolution course, to focus on areas where hospitalists identified deficits in this skillset. We presented a one-hour interactive workshop to physicians and advanced practice providers The workshop included didactics on negotiations and dispute resolution training with specific strategies, including anchoring, determining motivation, building trust, avoiding reaction, and spin. We also included a role-playing session during which participants practiced the strategies they had just learned. One month after the workshop, we emailed participating hospitalists a survey about the workshop asking how they had applied their new knowledge and skills. The survey included the validated Patient-Reported Outcomes Measurement Information System (PROMIS) measure for self-efficacy [11], the validated Dutch Test for Conflict Handling (DUTCH) [12], as well as a series of open-ended questions asking whether or not they had used any information gained from the workshop.

### 2.3. Data Analysis

Survey were collected using Qualtrics software with exports into Excel for quantitative analysis. For the quantitative data, descriptive statistics and 2-sided *t*-tests were used to analyze the quantitative survey responses. For the open-ended responses, content and constant comparative techniques were used whereby two coders independently assessed participant responses for focal themes before convening to compare and compile their findings to create a preliminary list of categories and major themes [13]. Discrepancies were resolved through discussion; there were no cases in which the coders were unable to reach consensus. The coders then organized the content into an overarching categorical system and agreed on themes that were particularly relevant. With qualitative analysis, 7–12 interviews are considered sufficient for thematic saturation [14].

## 3. Results

Thirty-two hospitalists attended the workshop with 19 (59.4%) Female, 26 (81.2%) Physician (MD/DO), and 6 (18.8%) Advanced Practice Providers (PA/NP). On average, attendees were 10.8 years post professional education with two also having a Master’s in Business Administration and two having a Master’s in Science. Nine hospitalists completed the pre-workshop and one month post-workshop surveys of which all reported experiencing conflicts with older adult patients at work.

### 3.1. Quantitative Results

Using the Dutch Test for Conflict Handling (DUTCH), participants self-categorized how they resolved a conflict at work into one of five dimensions: yielding, compromising, forcing, problem-solving, or avoiding [12]. In describing conflict personalities, subjects are usually a compilation of the ranges of each dimension (e.g., more yielding, more avoiding, less forcing). Prior to taking the workshop, as a group, subjects were high in yielding, compromising, and forcing attitudes. One month following the NDR workshop, there were trends in how subjects’ conflict resolution changed. Conflict avoidance, forcing, and yielding had decreased; while, problem solving behaviors increased (Table 1). None of the subcategories were statistically significant. Statistical analysis of pooled results from subcategories did not show significance in changes in conflict behaviors before and after the intervention (*p* = 0.31).

Using the PROMIS General Self-Efficacy short form, we asked participants to rate their confidence and self-efficacy in handling difficult problems and unexpected events [11]. Comparing baseline and 1 month post NDR workshop, there was significant improvement in self-efficacy (Baseline 14.26, 1 month post 15.67; *p* < 0.002).

### 3.2. Qualitative Results

Themes from the post-workshop survey responses are detailed in Table 2. The subthemes about encountered conflicts were (a) safe disposition after discharge (e.g., skilled nursing facility, caregiver support), (b) plans of care, (c) family request for 1:1 supervision while inpatient, (d) palliative care/hospice referrals.

We asked the participants open-ended questions one month after the workshop to explore the impact of the workshop on both their professional and personal lives. Six out of nine (67%) respondents reported that they had already used the skills learned during NDR training in either their professional and personal lives, and seven out of nine (78%) of respondents indicated interest in undergoing additional training. Table 2 highlights themes identified from the participants’ responses with representative quotes.

## 4. Discussion

Our results show that negotiations and dispute resolution workshop can benefit hospitalists in resolving conflicts. Participants in our workshop reported feeling empowered, with more self-efficacy, and confident in handling conflicts with their hospitalized older patients, which may increase the likelihood of patients receiving the social services they need.

Results from the one month follow-up revealed that NDR training had an impact on hospitalist clinical practice. Hospitalists reported being able to effectively negotiate with patients and their families by practicing the skills learned during the NDR training. Previous conflicts with older adults would have resulted in an impasse or in acquiescing to patients’ refusal of care. Instead, hospitalists were able to negotiate with patients and families, practicing the strategies learned in the workshop. We recognize that there was no significant difference in conflict behaviors before and after the workshop given the small numbers and short follow-up. However, both the qualitative results and positive trend in reported self-efficacy are encouraging and suggest that with additional training over a longer duration, we will likely see definitive changes in conflict behaviors.

This study shows that the skill set of negotiations and dispute resolution is one that is needed in hospitalists’ clinical practice, and they recognize its value in both their personal and professional lives. The benefits of NDR training for workshop participants extended beyond their clinical practice, into their personal lives. Hospitalists reported incorporating the negotiation and dispute resolution strategies into their personal lives and using those skills in negotiating chores at home as well as in completing personal purchases. This study had limitations, including it being conducted at a single site, the low response rate among all the attendees, and lack of understanding on direct effects on patients. These limitations provide areas for further study including the impact on older adult clinical outcomes. Nevertheless, we are encouraged by the findings and look forward to the next steps in the NDR training of clinicians to impact patient care.

Negotiation and dispute resolution training can be helpful to hospitalists who experience conflicts while caring for older adult patients. All of the surveyed hospitalists reported experiencing conflicts, with a common conflict occurring when older adults refuse social support assistance at discharge. Older adults have social needs but may not always be receptive to services that would fulfill these needs. Hence, it is imperative that clinicians caring for older adults at this vulnerable time are equipped with the knowledge and skills needed to overcome this refusal of services—knowledge and skills that NDR training provide.

## Figures and Tables

**Table 1:** Changes in negotiation skills post negotiations and dispute resolution (NDR) workshop.

	Baseline	1 Month Post
**DUTCH Subcategories**		
**Yielding**	14 (high)	13 (medium)
(high 14–20, medium 9–13, low 4–8)		
**Compromising**	14 (high)	13 (medium)
(14–20 high, 9–13 medium, 4–8 low)		
**Forcing**	14 (high)	11 (medium)
(4–8 low, 9–14 medium, 15–20 high)		
**Problem Solving**	16 (medium)	16 (high)
(high 17–20, medium 11–16, low 4–10)		
**Avoiding**	12 (medium)	11 (medium)
(low 4–7, medium 8–12, high 13–20)		

**Table 2:** Themes resulting from post NDR training responses.

Theme	Representative Quote(s)
NDR Training is Valuable	*“Gaining expertise in negotiations is a very useful life skill.”*
Conflicts with older adults regarding care plans is common.	*“They do not want a caregiver and fight it. The families are in conflict with them.”*
NDR training increases confidence in handling conflict	*“I feel more confident in dealing with conflict and talking options with seniors. I am not so stressed about dealing with conflict.”*
	*“Yes. It has made me feel more calm and thoughtful. It feels like there are more zen ways of handling conflict.”*
Increased Empowerment	*“It has empowered me to stop avoiding conflict.”*
	*“I hate conflict. The most valuable has been that it has allowed me to understand where I am as far as conflict and given me concrete ways of handling it…”*
Benefits Beyond Clinical Practice	*“Negotiating chores with partner.”*
	*“Yes. I have used it talking to colleagues when there has been a conflict. What will remedy this? What is important to you? The questioning part and asking for their issues and expanding the pie has been really helpful.”*
